# Peripheral Blood Transcriptomic Profile Associated with Cutaneous Radiation Injury in Cancer Patients Undergoing Radiotherapy

**DOI:** 10.3390/ijms27188168

**Published:** 2026-09-14

**Authors:** Seid Muhie, Nabarun Chakraborty, Mital Patel, Alison Ross, Lauren Moffatt, Melissa McLawhorn, Aarti Gautam, Jeffrey Shupp, Rasha Hammamieh

**Affiliations:** 1 Integrative Systems Biology, Center for Military Psychiatry and Neuroscience, Walter Reed Army Institute of Research, Silver Spring, MD 20910, USA; smuhie@genevausa.org (S.M.); nabarun.chakraborty2.civ@health.mil (N.C.); mital.y.patel.ctr@health.mil (M.P.); aarti.gautam.civ@health.mil (A.G.); 2 The Geneva Foundation, Silver Spring, MD 20910, USA; 3 Firefighters’ Burn and Surgical Research Laboratory, MedStar Health Research Institute, Washington, DC 20010, USA; alison.r.ross@medstar.net (A.R.); lauren.t.moffatt@medstar.net (L.M.); melissa.m.mclawhorn@medstar.net (M.M.); jeffrey.w.shupp@medstar.net (J.S.); 4 Department of Surgery, Georgetown University, Washington, DC 20007, USA; 5 Department of Biochemistry, Georgetown University, Washington, DC 20057, USA; 6 The Burn Center, Department of Surgery, MedStar Washington Hospital Center, Washington, DC 20010, USA

**Keywords:** cutaneous radiation injury, acute radiation dermatitis, peripheral blood transcriptomics, fractionated radiotherapy, radiation response, gene-set enrichment analysis

## Abstract

The peripheral blood transcriptomic profile associated with clinically observed cutaneous radiation injury (CRI) during fractionated radiotherapy remains incompletely characterized. We analyzed blood transcriptomes from 22 adults receiving radiotherapy: 10 with CRI and 12 without radiation-related skin injury at blood collection. RNA was assayed using two-color Agilent microarrays. Variance filtering retained 12,251 probes that were evaluated using four complementary analyses: preranked Gene-Set Enrichment Analysis (GSEA) for ranked gene-set enrichment, Gene-Set Variation Analysis (GSVA) for sample-level gene-set scoring, weighted gene co-expression network analysis for co-expression structure, and differential expression analysis at the probe level. GSEA identified positive enrichment of interferon-γ response and E2F targets and negative enrichment of heme metabolism. Consistently, GSVA showed higher interferon-response, E2F-target, unfolded-protein-response, and cholesterol-homeostasis scores and lower angiogenesis, xenobiotic-metabolism, protein-secretion, hypoxia, and heme-metabolism scores in participants with CRI. Ten modular co-expression networks met the data-derived CRI-correlation prioritization criterion. Positively correlated networks were enriched for negative regulation of response to wounding and complement/coagulation cascades. Intramodular connectivity and sample-level expression analyses prioritized KDM4C and PARPBP as exploratory high-connectivity candidates, with both retaining top-10 connectivity in 95.5% of leave-one-out iterations. Exploratory differential expression analysis identified 1842 probes, comprising 585 upregulated and 1257 downregulated probes in CRI. Genes mapped from downregulated probes were significantly enriched for ribosome, spliceosome, and oxidative phosphorylation pathways. Across complementary analytical frameworks, CRI was associated with a coherent peripheral blood profile characterized by interferon and cell-cycle enrichment; altered wound-response and complement/coagulation processes; reduced heme-metabolism, angiogenesis, and xenobiotic-metabolism scores; and downregulated transcripts involved in ribosomal function, RNA processing, and mitochondrial energy metabolism. Although the cross-sectional design and later sampling of CRI participants precluded full separation of CRI-associated differences from treatment-time and cumulative-exposure effects, these findings extend the molecular characterization of CRI and identify pathways and high-connectivity candidates for longitudinal assessment of reproducibility, temporal dynamics, and potential diagnostic or predictive relevance.

## 1. Introduction

Cutaneous radiation injury (CRI), often manifesting clinically as acute radiation dermatitis, is a common complication of fractionated radiotherapy [1,2,3]. Clinical manifestations range from erythema, edema, and dry desquamation to moist desquamation and, in severe cases, ulceration or necrosis [2,3,4]. The occurrence and severity of acute skin injury vary with treatment technique, dose distribution, fractionation, and patient-level factors [3,5,6].

The biological basis of CRI involves interacting epithelial, stromal, vascular, and immune responses [2,7,8]. Ionizing radiation induces DNA damage and reactive oxygen species in keratinocytes, fibroblasts, endothelial cells, and other skin-resident populations, leading to inflammatory signaling, impaired epithelial renewal, vascular dysfunction, barrier disruption, and delayed tissue repair [2,7,9,10]. Endothelial activation and dysfunction after irradiation can promote inflammatory and procoagulant tissue states [10,11], while complement activation has also been implicated in radiation-associated tissue injury [12]. Clinically apparent CRI therefore reflects direct cellular damage within irradiated skin together with coordinated inflammatory, vascular, hemostatic, immune-regulatory, and tissue-repair responses [2,8].

Ionizing radiation can also produce systemic molecular changes detectable in peripheral blood. Blood gene-expression studies have demonstrated dose- and time-dependent transcriptional responses involving DNA-damage signaling, p53-associated responses, immune and inflammatory regulation, cell-cycle control, apoptosis, and cellular stress [13,14,15,16]. Studies of blood RNA integrity and whole-blood gene expression further indicate that circulating molecular profiles can reflect radiation exposure and post-irradiation outcomes in human and large-animal models [17,18]. Most blood-based radiation-response studies, however, have focused on biodosimetry, exposure classification, dose estimation, or broader radiation-injury outcomes [14,15,16,17,18]. Prospective associations of pretreatment ferritin, high-sensitivity C-reactive protein, and CD3-positive T-cell proportions with severe acute radiation dermatitis indicate that baseline systemic inflammatory and immune features may be related to cutaneous injury risk [6]. Nevertheless, peripheral blood transcriptomic features associated specifically with clinically observed CRI during fractionated radiotherapy remain poorly characterized.

Transcriptome-wide profiling provides a systems-level framework for addressing this knowledge gap. Differential expression analysis identifies transcript-level associations; weighted gene co-expression network analysis identifies modules of co-expressed genes associated with clinical traits [19]; Gene-Set Enrichment Analysis evaluates coordinated enrichment across ranked genes [20]; and Gene-Set Variation Analysis estimates sample-level variation in predefined gene sets [21]. These complementary approaches can reveal coordinated biological relationships that are not apparent from individual transcripts alone.

In this study, we profiled peripheral blood transcriptomes from patients undergoing fractionated radiotherapy who had clinically observed CRI and from those without observable radiation-related skin injury at blood collection. By integrating preranked gene-set enrichment, sample-level pathway scoring, weighted gene co-expression network analysis, connectivity-based candidate prioritization, and differential expression analysis, we aimed to characterize the transcriptomic profile associated with clinically observed CRI. The broader objective was to identify biological pathways and processes associated with this profile, and thereby inform mechanistic hypotheses about the peripheral blood transcriptional milieu accompanying CRI.

## 2. Results

### 2.1. Analytic Sample and Sampling Context

Peripheral blood transcriptomes were analyzed from 22 participants: 10 with clinically observed CRI and 12 without observable radiation-related skin injury at blood collection (Table 1). No demographic or background clinical characteristic, including age, sex, race, or cancer type, and no complete blood count measure met the descriptive imbalance-screening threshold of unadjusted *p* ≤ 0.10 (Table 1). Under the patient-care-first protocol, blood specimens from participants with clinically apparent CRI were collected as proximal to diagnosis as possible, whereas controls could be enrolled at any time during radiotherapy. Participants with CRI were therefore sampled later after radiotherapy initiation than controls. For the descriptive sampling-time summary, one CRI participant with a non-comparable 491-day interval was excluded; this participant was retained in all other analyses, including transcriptomic profiling. Among the remaining nine CRI participants, the median sampling time was 34.0 days, compared with 17.0 days in controls (*p* = 0.002; Table 1). The corresponding descriptive ranges were 14–58 days for CRI and 8–23 days for controls. Considering all 10 CRI participants, only 2 were sampled within the control-group timing range. Given the close relationship between treatment timing and CRI ascertainment and the limited temporal overlap between groups, the extent to which the observed transcriptomic differences were associated with CRI status versus treatment time could not be robustly determined.

### 2.2. Gene-Set Enrichment Analyses

#### 2.2.1. Preranked Gene-Set Enrichment Analysis

**Figure 1:**
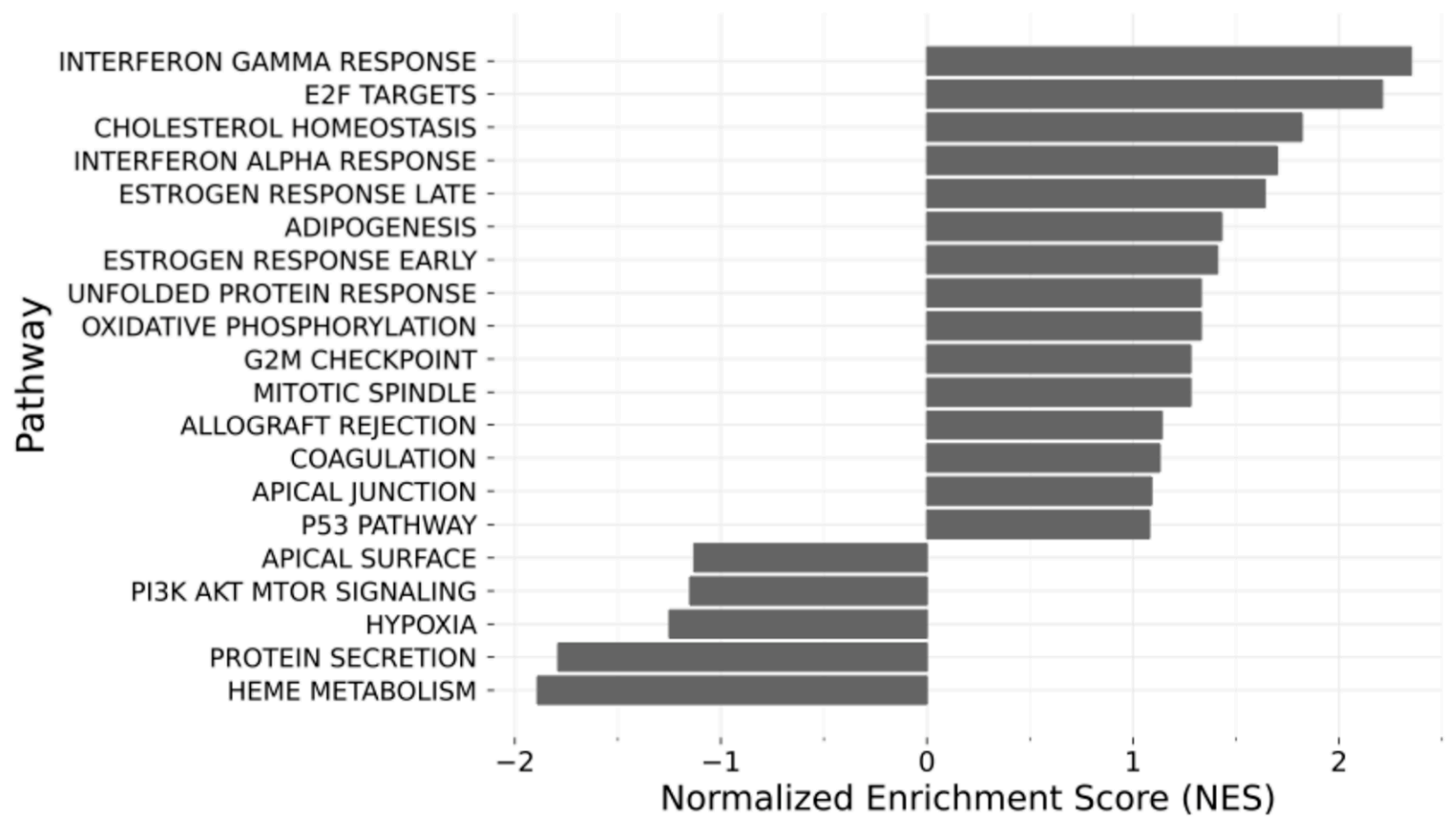
Hallmark gene sets identified by preranked Gene-Set Enrichment Analysis. The 20 MSigDB Hallmark gene sets with the smallest FDR-adjusted *p*-values are shown. Bars represent normalized enrichment scores. Positive scores indicate enrichment toward upregulated transcripts in CRI, whereas negative scores indicate enrichment toward downregulated transcripts.

Preranked Gene-Set Enrichment Analysis (GSEA) identified broad differential enrichment of MSigDB Hallmark gene sets in CRI relative to controls (Figure 1). The strongest positive enrichment was observed for interferon gamma response (normalized enrichment score [NES] = 2.45; FDR-adjusted *p* = 1.03 × 10^−2^), followed by E2F targets (NES = 2.21; FDR-adjusted *p* = 1.03 × 10^−2^). Other positively enriched gene sets included interferon alpha response, cholesterol homeostasis, estrogen-response programs, adipogenesis, unfolded protein response, oxidative phosphorylation, and cell-cycle-related programs. The strongest negative enrichment was observed for heme metabolism (NES = −1.90; FDR-adjusted *p* = 5.24 × 10^−3^) followed by protein secretion, hypoxia, PI3K–AKT–mTOR signaling, and apical-surface gene sets. Collectively, the enrichment profile was characterized by coordinated differences in immune-response, proliferative, metabolic, and cellular-stress programs in participants with clinically observed CRI.

**Figure 2:**
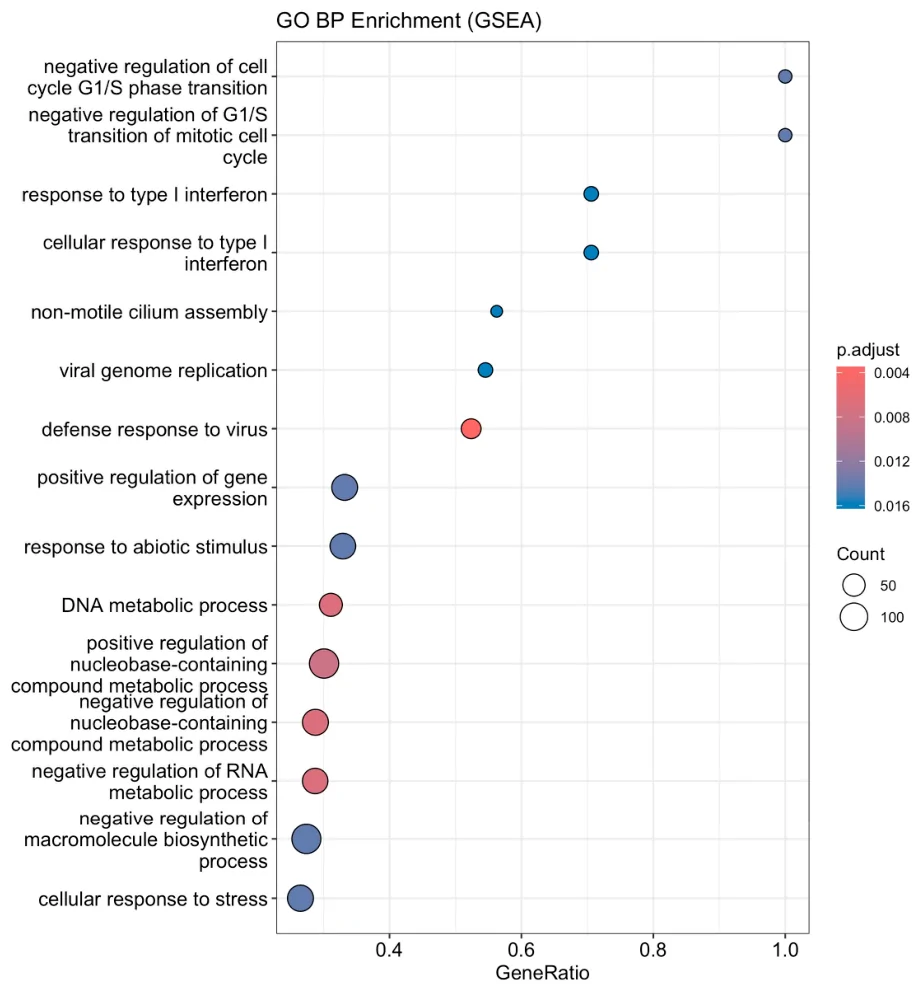
Gene Ontology Biological Process terms identified by ranked-profile Gene-Set Enrichment Analysis (GSEA). The same gene-symbol-ranked profile of moderated *t*-statistics used for the Hallmark GSEA was analyzed using clusterProfiler::gseGO() for Gene Ontology Biological Process terms. The *x*-axis shows GeneRatio, point size indicates the number of core-enrichment genes, and color indicates the adjusted *p*-value.

Complementary GSEA of Gene Ontology Biological Process terms using the same ranked transcriptomic profile identified enrichment of processes related to cell-cycle regulation, type I interferon responses, antiviral defense, DNA and RNA metabolism, biosynthetic regulation, and cellular stress (Figure 2). Among the displayed terms were negative regulation of the cell-cycle G1/S-phase transition, negative regulation of the G1/S transition of the mitotic cell cycle, response to type I interferon, cellular response to type I interferon, defense response to virus, DNA metabolic process, negative regulation of the RNA metabolic process, negative regulation of the macromolecule biosynthetic process, and cellular response to stress.

#### 2.2.2. Gene-Set Variation Analysis

**Figure 3:**
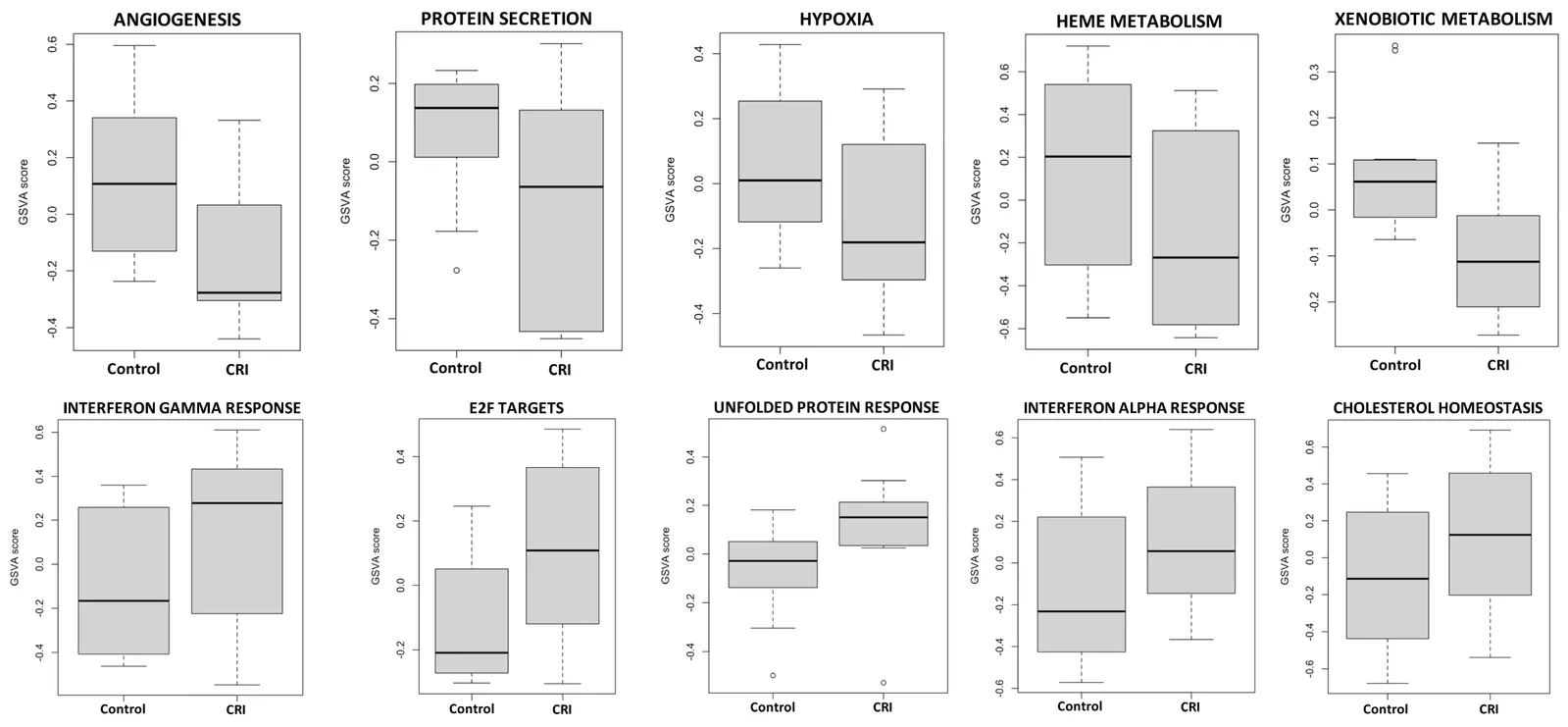
Sample-level Gene-Set Variation Analysis scores for Hallmark gene sets in CRI and control participants. The upper row shows gene sets with lower scores in CRI, and the lower row shows gene sets with higher scores in CRI. Boxes indicate medians and interquartile ranges; whiskers extend to the extreme observations within 1.5 times the interquartile range, and points denote observations beyond the whiskers. Higher GSVA scores indicate greater relative enrichment of the constituent genes.

Sample-level Gene-Set Variation Analysis (GSVA) scores were lower in CRI for angiogenesis (−0.308), heme metabolism (−0.28), xenobiotic metabolism (−0.18), protein secretion (−0.17) and hypoxia (−0.14) (Figure 3). Positive shifts were observed for interferon gamma response (0.24), interferon alpha response (0.23), E2F targets (0.22), cholesterol homeostasis (0.22), and unfolded protein response (0.19) (Figure 3). These profiles were directionally concordant with preranked GSEA findings such as interferon responses, E2F targets, heme metabolism, unfolded protein response, cholesterol homeostasis, protein secretion, and hypoxia.

### 2.3. Co-Expression Network Analysis

**Figure 4:**
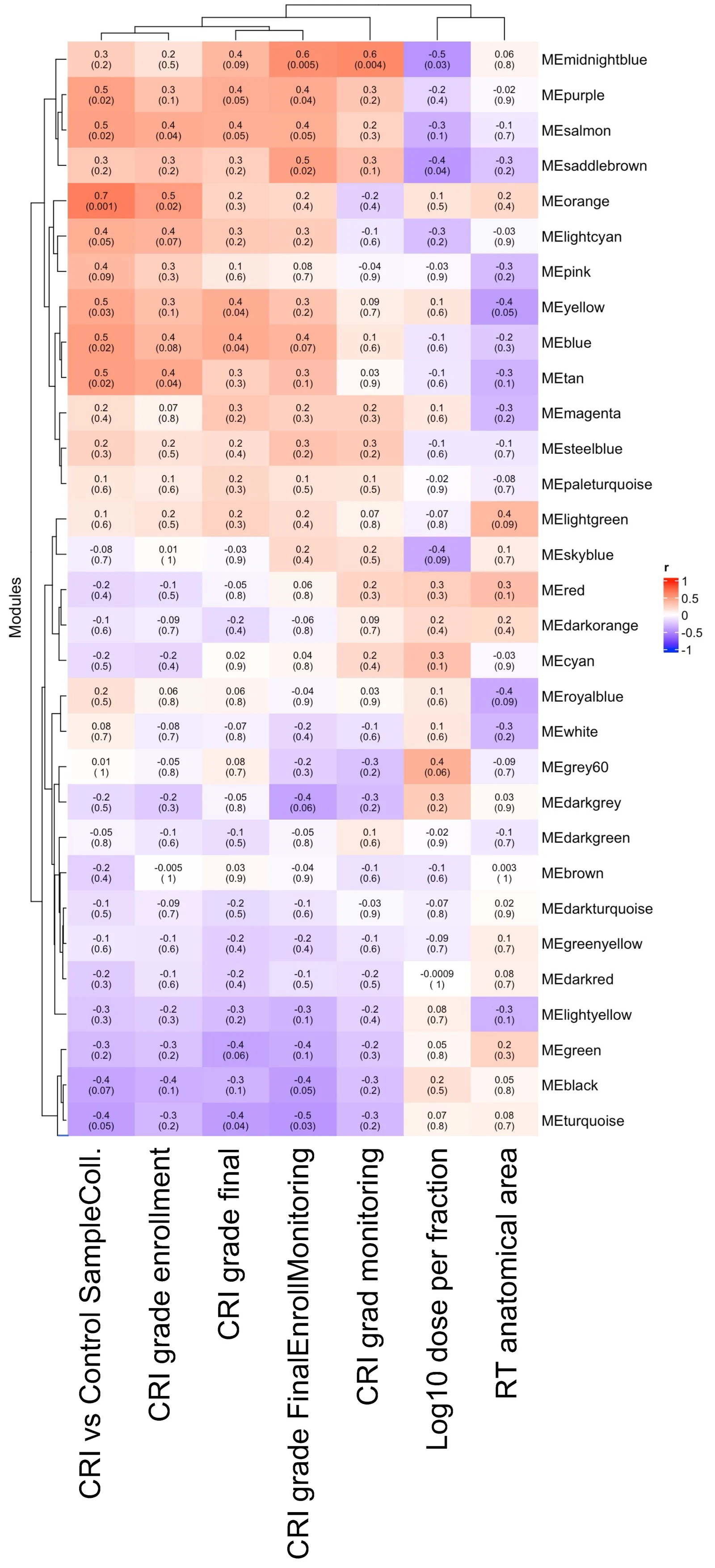
Correlation between weighted gene co-expression network modules and cutaneous radiation injury status. Biweight midcorrelations between module eigengenes and CRI status at blood collection are shown. Positive correlations indicate higher module eigengene values in participants with CRI, whereas negative correlations indicate higher values in controls. Module colors correspond to the WGCNA module assignments.

Weighted gene co-expression network analysis identified 10 modules that met the data-derived CRI-correlation prioritization criterion based on the absolute module–CRI correlation (Figure 4). Eight modules—orange, blue, tan, salmon, purple, yellow, lightcyan, and pink—were positively associated with CRI, with biweight midcorrelations ranging from 0.4 to 0.7 and *p* < 0.10. The black and turquoise modules were negatively correlated (−0.4) with CRI at *p* < 0.10.

Transcript members of positively associated modules were enriched for negative regulation of response to wounding (GO:0035890; FDR-adjusted *p* = 0.010) and complement and coagulation cascades (hsa04610; FDR-adjusted *p* = 0.018). Negatively correlated modules showed suggestive enrichment for extracellular matrix–receptor interaction (hsa04512; FDR-adjusted *p* = 0.081) and motor proteins (hsa04814; FDR-adjusted *p* = 0.157).

Connectivity-based analysis identified probe A_23_P112201, mapping to KDM4C in the yellow module, and probe A_23_P87769, mapping to PARPBP in the blue module, as exploratory high-connectivity candidates. In the full cohort, the KDM4C probe ranked first within the yellow module and the PARPBP probe ranked fourth within the blue module by intramodular topological-overlap connectivity. Sample-level expression profiles of the top-connectivity probes were additionally examined as a secondary qualitative prioritization step. This assessment supported prioritization of KDM4C and PARPBP based on the combination of high intramodular connectivity and cohort-level expression pattern rather than connectivity rank alone.

In participant-level leave-one-out analysis, both candidates retained top-10 intramodular connectivity in 21 of 22 iterations (95.5%). The PARPBP-containing blue module met the data-derived CRI-correlation prioritization criterion in all 22 iterations, resulting in joint retention of the connectivity and module-prioritization criteria in 21 of 22 iterations (95.5%). The KDM4C-containing yellow module met the data-derived CRI-correlation prioritization criterion in 14 of 22 iterations (63.6%), resulting in joint retention in 14 of 22 iterations. The direction of the module–CRI correlation remained unchanged in all 22 leave-one-out iterations for both modules. These results indicate greater overall participant-level stability for PARPBP. KDM4C showed similarly high retention of its intramodular connectivity ranking, but CRI prioritization of the KDM4C-containing yellow module was more sensitive to individual observations.

### 2.4. Differential Expression Analysis

**Figure 5:**
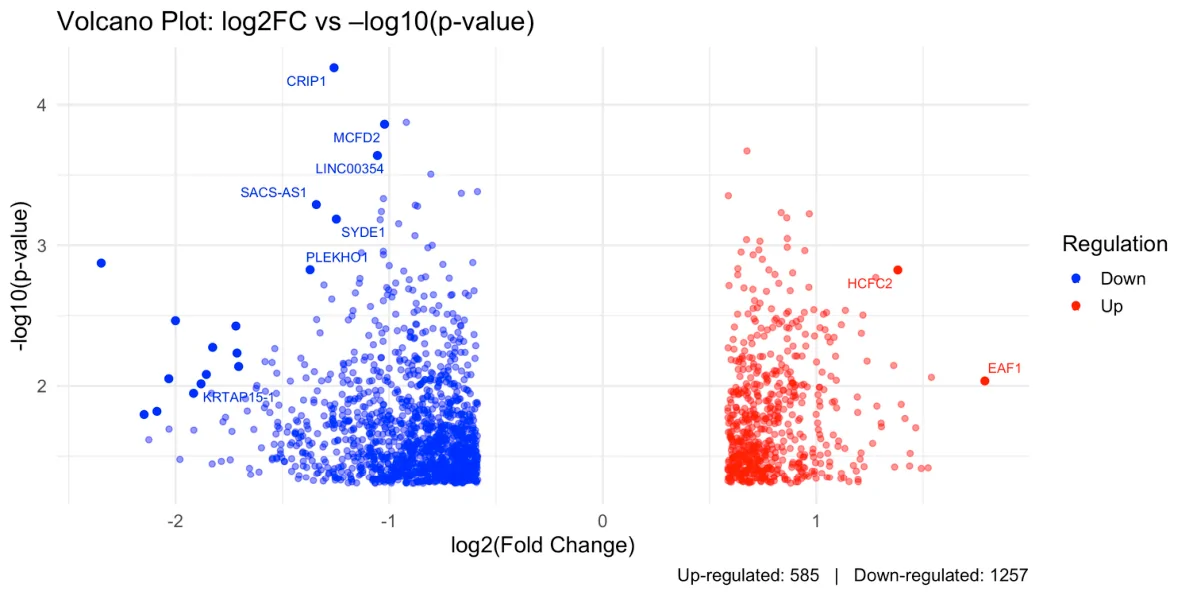
Exploratory probe-level expression differences between CRI and control participants. Probes meeting the exploratory criteria of unadjusted *p* < 0.05 and absolute linear fold change > 1.5 are shown according to the direction of the expression difference. No individual probe met FDR-adjusted *p* < 0.05.

Differential expression analysis comparing participants with CRI and controls identified 1842 probes meeting the exploratory criteria of unadjusted *p* < 0.05 and absolute linear fold change > 1.5. Of these, 585 showed higher expression and 1257 showed lower expression in participants with CRI relative to controls (Figure 5). Benjamini–Hochberg adjustment was applied across all 12,251 variance-filtered probes, and no individual probe met FDR-adjusted *p* < 0.05. The probe-level findings were therefore interpreted as exploratory and were used for direction-specific functional enrichment rather than as individually FDR-significant transcriptomic alterations.

#### Functional Enrichment of Genes Mapped from Exploratory Differential Expression Probe Sets

Direction-specific over-representation analysis was performed on genes mapped from the exploratory probe sets. Probes with lower expression in CRI mapped to 333 unique Entrez gene identifiers and showed strong pathway-level over-representation for ribosome (hsa03010; FDR-adjusted *p* = 4.8 × 10^−40^), spliceosome (hsa03040; FDR-adjusted *p* = 2.2 × 10^−27^), and oxidative phosphorylation (hsa00190; FDR-adjusted *p* = 4.5 × 10^−14^). These FDR-adjusted *p*-values refer to pathway-level over-representation and do not indicate the FDR significance of the individual constituent probes. Probes with higher expression in CRI mapped to 134 unique Entrez gene identifiers and showed suggestive enrichment for negative regulation of response to wounding (GO:0035890; FDR-adjusted *p* = 0.052), negative regulation of wound healing (GO:0061046; FDR-adjusted *p* = 0.088), and negative regulation of blood coagulation (GO:0050819; FDR-adjusted *p* = 0.106).

### 2.5. Integrated Transcriptomic Profile Associated with CRI

Integration of preranked gene-set enrichment, sample-level pathway scoring, co-expression network analysis, and exploratory differential expression analysis identified a convergent peripheral blood transcriptomic profile associated with clinically observed CRI. This profile included positive enrichment of interferon-response and E2F-target gene sets; negative enrichment of heme-metabolism, protein-secretion, and hypoxia gene sets; lower angiogenesis and xenobiotic-metabolism scores; and downregulated transcripts involved in ribosomal, spliceosomal, and oxidative phosphorylation pathways. Co-expression analysis additionally implicated negative regulation of response to wounding and complement/coagulation processes. Intramodular connectivity and sample-level expression analyses prioritized KDM4C and PARPBP as exploratory high-connectivity candidates, with both showing stable top-10 connectivity in leave-one-out analysis; CRI prioritization was more consistently retained for the PARPBP-containing network. Taken together, the complementary analyses support coordinated pathway- and network-level associations involving interferon and cell-cycle signaling, wound-response and complement/coagulation processes, and ribosomal, RNA-processing, and mitochondrial energy functions, while individual probe- and candidate-gene findings remain exploratory.

## 3. Discussion

Clinically observed cutaneous radiation injury (CRI) during radiotherapy was associated with a coordinated peripheral blood transcriptomic profile involving immune and cell-cycle responses; wound repair and hemostasis; and biosynthetic, mitochondrial, metabolic, and angiogenesis-related alterations. This profile was evident across gene-set enrichment, sample-level pathway scoring, co-expression network analysis, and exploratory differential expression analysis. The convergence of these analytically distinct results indicates that the association was not driven by a single transcript or isolated pathway. Accordingly, clinically apparent CRI was accompanied by broad differences in circulating transcriptional activity. The principal biological inference is therefore not that CRI ceases to be a localized tissue injury, but that its clinical manifestation occurs alongside a measurable systemic host response.

Interferon-response enrichment was among the most consistent findings. Peripheral blood transcriptional responses to ionizing radiation are well established and vary with radiation dose and time after exposure, with recurrent changes in DNA-damage, inflammatory, and immune-response genes [13,14,16]. Experimental studies provide a plausible mechanistic connection between radiation-induced DNA damage and interferon-related transcription: damaged DNA entering the cytosol, including DNA exposed following micronuclear-envelope rupture, can activate cGAS–STING-dependent interferon responses [22,23,24]. These mechanisms support the biological plausibility of the interferon-related enrichment observed here, which is best interpreted as evidence of a systemic interferon-responsive transcriptional state accompanying clinically apparent CRI.

E2F-target enrichment represents a second component of the systemic profile associated with CRI. E2F-regulated genes integrate cell-cycle progression with DNA replication, checkpoint control, and DNA-damage responses [25]. In peripheral blood, this enrichment may reflect altered cell-cycle or checkpoint states within circulating cells, radiation-associated repair activity, or differences in responsive leukocyte subsets. The interferon and E2F findings indicate that the CRI-associated blood profile encompasses both immune-response and cell-state regulation rather than inflammation alone.

The wound-response and complement/coagulation findings provide the clearest biological link between the circulating profile and clinically apparent tissue injury. Positively correlated co-expression modules were significantly enriched for wound-response processes and complement and coagulation cascades. Radiation injury involves epithelial and endothelial damage, inflammatory-cell recruitment, vascular dysfunction, barrier disruption, and altered tissue repair, with extensive interactions among inflammatory, complement, coagulation, and vascular responses [8,10,11,12]. Prospective clinical evidence associating circulating inflammatory and immune features with acute radiation dermatitis severity further supports the relevance of systemic host state to cutaneous injury during radiotherapy [6]. These findings link clinically observed CRI to a peripheral blood transcriptional profile involving wound-response and complement/coagulation processes.

The dominant direction of the exploratory differential expression results was downregulation in CRI. Lower-expression probes were strongly enriched for ribosome, spliceosome, and oxidative phosphorylation pathways, while complementary analyses showed negative heme-metabolism enrichment and lower xenobiotic-metabolism and angiogenesis scores. Together, these findings indicate lower biosynthetic and RNA-processing signals, together with lower heme-metabolism, xenobiotic-metabolism, and angiogenesis-related signals. Oxidative-phosphorylation-related findings differed across analytical frameworks: Hallmark GSEA, which evaluates enrichment across the full ranked transcriptomic profile, showed positive enrichment in CRI, whereas KEGG over-representation analysis identified oxidative phosphorylation among genes mapped from the exploratory lower-expression probe set. This difference may reflect the distinct gene-set definitions and analytical inputs of the two approaches. Accordingly, the GSEA result is more directly informative regarding pathway-level directionality, whereas the over-representation result indicates that oxidative phosphorylation genes were disproportionately represented within the exploratory lower-expression set. These findings should therefore not be interpreted as evidence of a uniformly lower oxidative phosphorylation state in CRI.

Ionizing radiation can alter gene expression through translational control, changes in polysome-associated RNA, and alternative transcript usage [26,27], while inflammatory responses can remodel leukocyte biosynthetic and mitochondrial states [28,29]. These mechanisms provide a plausible biological context for the observed CRI-associated patterns of lower biosynthetic and selected metabolic signals, altered mitochondrial-energy-related signals, and higher immune- and injury-response expression.

Beyond the wound-response and complement/coagulation findings, co-expression analysis highlighted suggestive extracellular matrix–receptor interaction and motor-protein enrichment in negatively correlated modules. In peripheral blood, these patterns may reflect leukocyte adhesion, migration, cytoskeletal organization, or circulating-cell states rather than direct dermal matrix remodeling. The network analysis further prioritized KDM4C and PARPBP as exploratory high-connectivity candidates based on complementary connectivity and sample-level expression criteria. Leave-one-out sensitivity analysis provided stronger participant-level support for PARPBP, whereas prioritization of the KDM4C-containing yellow module was more sensitive to individual observations. KDM4C is a histone lysine demethylase involved in chromatin regulation and has been linked experimentally to DNA-damage responses and radiosensitivity-related phenotypes [30,31], whereas PARPBP/PARI is a PCNA-associated regulator of homologous recombination acting through PCNA- and RAD51-related repair mechanisms [32,33]. These biological functions provide a rationale for further investigation, but both candidates remain exploratory and require independent validation.

Previous peripheral blood transcriptomic studies have largely focused on radiation exposure and biodosimetry. The present study extends this work by associating clinically observed normal-tissue injury during fractionated radiotherapy with a genome-wide blood transcriptomic profile spanning immune and cell-state regulation; wound repair and hemostasis; and biosynthetic, mitochondrial, metabolic, and angiogenesis-related alterations. Longitudinal studies integrating serial transcriptomics with skin dosimetry, clinical grading, cell-resolved immune profiling, and paired tissue or protein measurements are needed to distinguish between susceptibility, cumulative exposure, evolving injury, and recovery, and to determine whether selected blood-based features can support risk stratification or objective monitoring.

The study’s modest cohort size and cross-sectional design, together with later sampling of participants with CRI and limited temporal overlap between groups, prevented robust separation of CRI-associated transcriptomic differences from treatment-time and cumulative-exposure effects. Because sampling time was closely linked to CRI ascertainment, the observed molecular differences should be interpreted within their treatment and sampling context rather than as CRI-specific effects independent of those factors. More detailed radiotherapy characteristics relevant to local cutaneous exposure—including treatment technique, bolus use, boost irradiation, beam arrangement, and patient-level skin-dose, hot-spot, and dose-volume metrics—were not consistently available, limiting assessment of the contribution of local treatment factors to the observed clinical phenotype and peripheral blood associations. The limited CRI severity range also precluded reliable severity-resolved transcriptomic analysis. Bulk peripheral blood profiling could not resolve the specific cellular sources of the observed transcriptomic differences. These findings therefore require confirmation in larger longitudinal cohorts with prespecified serial sampling, detailed and harmonized dosimetry, broader representation of CRI severity, cell-resolved profiling, and independent molecular validation.

## 4. Materials and Methods

### 4.1. Study Design, Participant Status and Analytic Sample

Twenty-two adults receiving radiotherapy for breast (n = 20) or laryngeal (n = 2) cancer were prospectively enrolled in this observational study after providing informed consent. Participants were clinically evaluated for observable radiation-related skin injury at peripheral blood collection. Those with clinically observed cutaneous radiation injury (CRI) were classified as CRI cases, and sex- and cancer type–matched participants without observable injury were classified as controls. Participants were followed for up to 4 weeks after sampling. Clinical status at the time of blood collection defined the molecular comparison; subsequent development of dermatitis did not alter the classification of an already assayed specimen because no corresponding transcriptomic specimen was collected at the later onset. Accordingly, the molecular analyses addressed the peripheral blood transcriptomic profile associated with clinical status at the time of sampling rather than subsequent susceptibility or prediction. Analyses were conducted at the participant level, and the final analytic cohort comprised 10 participants with CRI and 12 controls.

### 4.2. Radiotherapy and CRI Assessment

Radiotherapy was delivered per standard of care. Among the 20 participants receiving breast or chest-wall irradiation, the recorded treatment field was breast in 16 and chest wall in 4; six treatments included regional lymphatic regions. Eight participants received 42.56 Gy in 16 fractions (2.66 Gy/fraction), and 12 received 50.40 Gy in 28 fractions (1.80 Gy/fraction). Two participants received laryngeal or head-and-neck irradiation using 63.00 Gy in 28 fractions (2.25 Gy/fraction; n = 1) or 54.00 Gy in 30 fractions (1.80 Gy/fraction; n = 1). More detailed radiotherapy characteristics relevant to local cutaneous exposure—including treatment technique, bolus use, boost irradiation, beam arrangement, and patient-level skin-dose, hot-spot, or dose-volume metrics—were not consistently available across the cohort and therefore could not be systematically evaluated in the present analyses. The available treatment-site records supported classification as breast or chest wall but did not permit a consistently verified surgical-status classification beyond these treatment-site labels.

Radiation dermatitis was graded using the National Cancer Institute Common Terminology Criteria for Adverse Events v5.0. CRI required Grade ≥ 1 documented within 7 days before blood collection. At collection, CRI cases had Grade 1 (n = 5) or Grade 2 (n = 5) dermatitis, whereas controls had no observable radiation-related skin injury. The primary transcriptomic comparison was binary CRI versus control. Because only five CRI participants had Grade 1 and five had Grade 2 dermatitis at blood collection, with no Grade ≥ 3 cases represented at the assayed time point, formal grade-stratified transcriptomic analyses were not performed. Because repeat sampling was unavailable for most participants, the molecular analyses were cross-sectional and classification reflected clinical status at blood collection.

### 4.3. Variable Handling and Preprocessing

Demographic, treatment, and complete blood count (CBC) variables were extracted from the case report table. The primary grouping variable was analytic status, defined as CRI versus control. Age was converted from source units to years for interpretability. Race checkbox fields were harmonized into a single mutually exclusive categorical variable with the following levels: Caucasian, Black, Other, and Unknown. Missing data were handled using available-case analysis for each variable, without imputation. For continuous variables, the non-missing sample size was reported.

To reduce the risk of causal-role misclassification, variables were prespecified according to their temporal and biological roles. Variables were classified as (i) pre-treatment or background variables, considered potential baseline covariates; (ii) treatment- and sampling-context variables, considered exposure-context descriptors and interpreted in relation to CRI ascertainment rather than used for routine molecular adjustment; and (iii) post-sampling variables, excluded from molecular adjustment because they occurred after blood collection and could lie downstream of treatment- or CRI-related processes. Radiation dermatitis grade at blood collection was not included as an adjustment covariate because it was directly related to the CRI phenotype definition.

### 4.4. Statistical Analysis and Between-Group Imbalance Screening

Participant-level demographic, clinical, complete blood count, and treatment- and sampling-context variables were summarized by CRI status (Table 1). Continuous variables were reported as the non-missing sample size, mean ± standard deviation, and median with interquartile range, and were compared using two-sided Mann–Whitney U tests. Categorical variables were reported as counts and percentages. Binary variables were compared using Fisher’s exact test; multilevel variables were evaluated using exact tests when feasible or chi-square tests when expected cell counts were adequate. Sparse comparisons were summarized descriptively without formal testing.

An unadjusted *p*-value threshold of ≤0.10 was used only to flag potential between-group imbalance for descriptive review. This threshold was not used to define confounding or to mechanically select covariates. Potential covariate relevance was evaluated according to temporal ordering, biological plausibility, and the covariate-role classification described above rather than on the basis of *p*-values alone. All tests were two-sided, with statistical significance defined as *p* < 0.05 unless otherwise specified.

### 4.5. Treatment and Sampling Context

The primary transcriptomic analyses compared participants according to CRI status at blood collection. Under the patient-care-first clinical protocol, patients continued to receive treatment according to the attending and clinical team’s standard of care, and no study-related intervention altered that care. Blood specimens from participants with clinically apparent CRI were collected as proximal to diagnosis as possible, whereas controls could be enrolled at any time during radiotherapy. Accordingly, specimen timing reflected the protocol-defined sampling framework and the occurrence and recognition of dermatitis rather than an experimental strategy designed to match CRI and control participants on time from radiotherapy initiation or cumulative radiation exposure.

Participants with CRI were sampled later during radiotherapy than controls, and the timing distributions showed limited overlap. Time from radiotherapy initiation to blood collection was therefore considered an important treatment- and sampling-context variable and a potential source of confounding because peripheral blood transcriptional responses to radiation can vary over time. Given the close relationship between sampling time and clinical CRI ascertainment, the small cohort, and the limited temporal overlap between groups, the extent to which the observed transcriptomic differences were associated with CRI status versus treatment time could not be robustly determined without substantial extrapolation. A comparable cumulative radiation dose at the exact time of blood collection was also not consistently reconstructable across participants. Accordingly, the transcriptomic findings were interpreted as cross-sectional associations with clinically observed CRI within the treatment and sampling context in which the blood specimens were obtained; no treatment-time or cumulative-dose adjustment was used to estimate an association between CRI status and transcriptomic outcomes independent of these treatment-context variables.

### 4.6. RNA Extraction, Microarray Assay, Quality Control, and Normalization

Total RNA from whole blood was extracted using PAXgene Blood RNA purification kits (QIAGEN LLC, Germantown, MD, USA) and assayed using Agilent Human Gene Expression v3 8 × 60K two-color microarrays (G4851C; Agilent Technologies, Santa Clara, CA, USA). Although specimens were collected individually during participant enrollment, all 22 study samples were subsequently processed together as a single study set under the same microarray laboratory workflow and procedures. Raw red- and green-channel intensities were processed in limma using normexp background correction with an offset of 50 [34], within-array loess normalization, and between-array quantile normalization [35]. These procedures addressed background, dye-related, and between-array distributional technical variation. Post-normalization array quality was assessed on the normalized M-value matrix using arrayQualityMetrics v3.58.0, including between-array distances, principal component analysis, boxplots, density plots, mean–variance dependence, and MA plots. The complementary quality-control assessments were considered collectively, and no array was classified as an outlier requiring exclusion from downstream analyses; all study arrays were retained. Variance filtering based on a loess trend and median absolute deviation retained 12,251 probes for downstream analyses.

### 4.7. Gene-Set Enrichment, Co-Expression Network, and Differential Expression Analysis

The normalized matrix of 12,251 variance-filtered probes was used for all downstream transcriptomic analyses without significance-based preselection. Gene-Set Enrichment Analysis (GSEA) used the complete ranked profile of moderated *t*-statistics. Probes lacking mapped gene symbols were excluded, and when multiple probes mapped to the same gene symbol, only the first occurrence in the ordered limma results was retained. For Gene-Set Variation Analysis (GSVA), normalized expression values from probes mapping to the same symbol were averaged to obtain one gene-level expression profile. Weighted gene co-expression network analysis (WGCNA) and differential expression analysis were conducted on the probe-level expression matrix.

Preranked GSEA was performed using moderated *t*-statistics from the CRI-versus-control limma contrast [20,36]. Hallmark gene-set enrichment was evaluated with fgsea against the Molecular Signatures Database Hallmark collection obtained through msigdbr [37], using 10,000 permutations and gene sets containing 10–500 represented genes. False-discovery-rate-adjusted *p*-values were calculated across Hallmark gene sets. In a complementary ranked-profile analysis, the same ranked gene-symbol list was evaluated for Gene Ontology Biological Process terms using clusterProfiler::gseGO() with keyType = “SYMBOL”, ont = “BP”, gene-set sizes of 10–500 genes, and *p*valueCutoff = 0.25. GSVA was applied to the gene-level expression matrix using the same Hallmark collection to estimate sample-level gene-set enrichment scores [21]. Scores were compared between CRI and control participants using a group-only limma model with empirical Bayes moderation and Benjamini–Hochberg adjustment [36].

WGCNA was performed on the normalized probe-level expression matrix [19,38]. The soft-thresholding power was selected from values 1–20 using scale-free-topology diagnostics, with 6 used as a fallback when no estimate was returned. Module detection used a minimum module size of 30, a merge cut height of 0.15, and TOMType = ‘signed’. Module eigengenes were associated with binary CRI status using biweight midcorrelation with pairwise-complete observations, and module–trait *p*-values were calculated using the Student correlation approximation [38].

Modules were prioritized when the absolute module–CRI correlation was at least the mean absolute module–CRI correlation across modules plus one standard deviation; the correlation sign indicated the direction of association. Intramodular connectivity (kWithin) was defined for each probe as the row sum of the within-module topological overlap matrix (TOM). Probes were ranked by kWithin, and the 10 highest-connectivity probes within each evaluated module defined the initial exploratory high-connectivity candidate set. Rank within this top-10 set was not used as the sole basis for final candidate prioritization. As a secondary qualitative prioritization step, sample-level expression profiles of these probes were visually examined to favor candidates whose patterns were broadly represented across participants and were not explained solely by isolated extreme observations. This combined assessment prioritized probe A_23_P112201 (which maps to KDM4C) in the yellow module and probe A_23_P87769 (which maps to PARPBP) in the blue module. The visual assessment refined candidate prioritization but was not treated as a formal statistical test of robustness.

Participant-level stability of KDM4C and PARPBP prioritization was evaluated using leave-one-out sensitivity analysis. The original module assignments and full-data soft-thresholding power were held fixed, and each participant was omitted sequentially. For each reduced dataset, the topological overlap matrix, intramodular connectivity, module eigengenes, and module–CRI biweight midcorrelations were recalculated. Stability was summarized by whether each candidate remained among the 10 highest-connectivity probes in its original module, whether that module met the data-derived CRI-correlation prioritization criterion, and whether the direction of the module–CRI correlation was preserved.

Functional enrichment analyses with Benjamini–Hochberg correction were conducted separately for prioritized positively and negatively CRI-correlated WGCNA modules.

Probe-level differential expression was evaluated using linear models with empirical Bayes moderation in limma [36]. The group-only design compared CRI with control participants. Benjamini–Hochberg false-discovery-rate-adjusted (*p*)-values were calculated across all 12,251 probes. Probes meeting nominal *p* < 0.05 and absolute linear fold change > 1.5 were mapped to Entrez gene identifiers and separated by expression direction. Gene Ontology biological-process and KEGG pathway over-representation analyses were conducted independently for genes with higher and lower expression in CRI.

### 4.8. Versions of R (v4.5.3) Packages Used for Statistical Analyses

Differential expression and GSVA comparisons were two-sided, and GSEA evaluated enrichment in both directions. Transcriptomic analyses used limma v3.66.0, arrayQualityMetrics v3.58.0, WGCNA v1.74, fgsea v1.36.2, GSVA v2.4.9, msigdbr v26.1.0, BiocParallel v1.44.0, and clusterProfiler v4.18.4. Supporting annotation and data processing used org.Hs.eg.db v3.22.0, AnnotationDbi v1.72.0, GSEABase v1.72.0, matrixStats v1.5.0, genefilter v1.92.0, dplyr v1.2.1, and ggplot2 v4.0.3.

## 5. Conclusions

This study identified a peripheral blood transcriptomic profile associated with clinically observed CRI during fractionated radiotherapy. The findings indicate that clinically apparent CRI is accompanied by a measurable systemic molecular response involving immune and cell-cycle responses; wound-response and hemostatic processes; and biosynthetic, RNA-processing, mitochondrial, metabolic, and angiogenesis-related functions. These findings extend the molecular characterization of CRI and provide a focused basis for longitudinal investigation. Serial blood sampling, detailed dosimetry, cell-resolved profiling, and independent validation will be important for establishing reproducibility and temporal relationships and for determining the potential diagnostic or predictive relevance of this circulating molecular profile. Such studies are also needed to distinguish CRI-associated differences from treatment-time and cumulative-exposure effects that could not be fully separated in the present cross-sectional study.

## Figures and Tables

**Table 1:** Participant-level analyses of demographic and clinical variables comparing CRI vs. controls.

Characteristic	CRI (n = 10)	Control (n = 12)	*p*-Value *
**Demographics**			
Age, years †	n = 10; 60.8 ± 14.4; 64.9 [54.5, 71.2]	n = 12; 58.6 ± 14.2; 62.2 [51.0, 68.7]	0.668
Sex as recorded			1.000
Female	9 (90.0%)	11 (91.7%)	
Male	1 (10.0%)	1 (8.3%)	
Race			0.602
Black	6 (60.0%)	9 (75.0%)	
Caucasian	3 (30.0%)	2 (16.7%)	
Other	1 (10.0%)	0 (0.0%)	
Unknown	0 (0.0%)	1 (8.3%)	
Ethnicity			0.571
Non-Hispanic	8 (80.0%)	11 (91.7%)	
Unknown	2 (20.0%)	1 (8.3%)	
Cancer type			1.000
Breast	9 (90.0%)	11 (91.7%)	
Laryngeal	1 (10.0%)	1 (8.3%)	
**Radiation treatment and timing**			
Days from RT start to initial sample collection	** n = 9; 34.8 ± 13.3; 34.0 [28.0, 38.0]	n = 12; 16.5 ± 5.8; 17.0 [10.8, 22.2]	0.002 §
Prescribed fractions, n	n = 10; 24.4 ± 5.8; 28.0 [19.0, 28.0]	n = 12; 23.2 ± 6.4; 28.0 [16.0, 28.0]	0.819
Dose per fraction, cGy	n = 10; 210 ± 41; 180 [180, 256]	n = 12; 216 ± 44; 180 [180, 266]	0.819
RT duration	n = 10; 86.3 ± 145.6; 37.5 [31.8, 49.5]	n = 10; 35.7 ± 7.3; 37.5 [30.5, 40.2]	0.383
Total dose at end of monitoring, cGy	n = 10; 8147 ± 3914; 6040 [5256, 10,830]	n = 12; 6472 ± 2758; 5256 [4950, 6840]	0.206
**Complete blood count (CBC)**			
WBC (k/µL)	n = 9; 5.76 ± 3.81; 4.40 [3.82, 5.35]	n = 12; 3.93 ± 1.42; 3.92 [2.81, 4.49]	0.241
Hemoglobin (g/dL)	n = 9; 11.9 ± 1.8; 12.5 [11.2, 12.5]	n = 12; 11.9 ± 1.3; 11.9 [11.1, 12.9]	1.000
Hematocrit (%)	n = 9; 37.1 ± 3.9; 38.3 [35.5, 39.2]	n = 12; 37.6 ± 3.6; 38.5 [34.8, 40.2]	0.831
Platelets (k/µL)	n = 9; 232 ± 54; 242 [231, 251]	n = 12; 224 ± 86; 240 [192, 264]	0.915
MCV (fL)	n = 9; 86.3 ± 6.0; 87.2 [84.1, 90.1]	n = 12; 87.5 ± 7.1; 87.0 [85.9, 89.0]	0.972
MCH (pg)	n = 9; 27.5 ± 2.9; 28.2 [27.4, 28.7]	n = 12; 27.8 ± 2.9; 28.1 [26.2, 29.1]	1.000
MCHC (g/dL)	n = 9; 31.8 ± 1.8; 31.9 [31.5, 32.6]	n = 12; 31.7 ± 1.1; 31.8 [30.6, 32.4]	0.643
RDW (%)	n = 9; 14.5 ± 1.9; 14.3 [13.2, 15.0]	n = 12; 14.4 ± 1.9; 14.2 [13.5, 15.1]	1.000
RBC (million/µL)	n = 9; 4.30 ± 0.42; 4.36 [4.00, 4.56]	n = 12; 4.31 ± 0.48; 4.40 [3.96, 4.62]	0.859
Neutrophils (%)	n = 8; 59.0 ± 12.5; 57.2 [47.0, 69.7]	n = 12; 58.9 ± 11.8; 62.6 [55.9, 64.7]	0.910
Lymphocytes (%)	n = 9; 22.9 ± 10.7; 18.0 [17.0, 31.8]	n = 12; 24.4 ± 11.6; 23.6 [18.1, 26.4]	0.670
Monocytes (%)	n = 9; 11.5 ± 4.4; 10.7 [9.1, 12.6]	n = 12; 12.3 ± 4.8; 10.9 [9.4, 13.5]	0.859
Eosinophils (%)	n = 9; 4.0 ± 4.7; 1.7 [1.2, 4.1]	n = 12; 3.3 ± 1.8; 3.9 [1.6, 4.4]	0.594
Basophils (%)	n = 9; 0.62 ± 0.36; 0.60 [0.30, 0.90]	n = 12; 0.70 ± 0.33; 0.70 [0.40, 0.95]	0.566
Absolute neutrophil count (k/µL)	n = 8; 3.49 ± 2.13; 2.80 [2.31, 4.53]	n = 12; 2.33 ± 1.09; 2.10 [1.53, 2.82]	0.262
Absolute lymphocyte count (k/µL)	n = 8; 1.59 ± 1.98; 0.75 [0.66, 1.55]	n = 12; 1.00 ± 0.55; 1.00 [0.65, 1.35]	1.000
Absolute monocyte count (k/µL)	n = 8; 0.64 ± 0.28; 0.50 [0.50, 0.65]	n = 12; 0.45 ± 0.13; 0.50 [0.38, 0.53]	0.149
Absolute eosinophil count (k/µL)	n = 8; 0.19 ± 0.17; 0.13 [0.06, 0.25]	n = 12; 0.13 ± 0.07; 0.10 [0.10, 0.20]	0.968
Immature granulocytes (%)	n = 8; 0.35 ± 0.12; 0.35 [0.28, 0.43]	n = 12; 0.37 ± 0.28; 0.40 [0.28, 0.40]	0.968
Absolute immature granulocyte count (k/µL)	n = 8; 0.02 ± 0.01; 0.01 [0.01, 0.02]	n = 12; 0.01 ± 0.01; 0.01 [0.01, 0.02]	0.300
MPV (fL)	n = 9; 10.1 ± 1.0; 9.9 [9.5, 10.6]	n = 12; 10.3 ± 0.9; 10.2 [10.1, 10.9]	0.670

Values are presented as n; mean ± SD; median [IQR] for continuous variables and n (%) for categorical variables. Missing data were handled using available-case analysis; per-variable non-missing sample size is shown. Raw *p* ≤ 0.10 was used only to flag potential between-group imbalance for descriptive review and was not used to define confounding or mechanically select covariates. Abbreviations: CRI, cutaneous radiation injury; RT, radiation treatment; IQR, interquartile range; SD, standard deviation; CBC, complete blood count; MPV, mean platelet volume. * Continuous variables were compared using two-sided Mann–Whitney U tests; binary categorical variables using Fisher’s exact test; and multilevel categorical variables using exact tests when feasible or chi-square tests when expected cell counts were adequate. ** For the descriptive sampling-time summary, one CRI participant with a non-comparable 491-day interval was excluded; this participant was retained in all other analyses, including transcriptomic profiling. † Age converted from source values to years for interpretability. § Meets imbalance screening threshold (raw *p* ≤ 0.10); this is descriptive and does not by itself establish causal confounding.

## Data Availability

Datasets are deposited at the Gene Expression Omnibus (GEO): accession GSE336147.

## Abbreviations

The following abbreviations are used in this manuscript:

CRI	Cutaneous radiation injury
GO	Gene Ontology
GSEA	Gene-Set Enrichment Analysis
GSVA	Gene-Set Variation Analysis
hsa	*Homo sapiens* (KEGG organism code)
KEGG	Kyoto Encyclopedia of Genes and Genomes
limma	Linear Models for Microarray Data (R/Bioconductor package)
NES	Normalized Enrichment Score
normexp	Normal–exponential convolution background correction
WGCNA	Weighted Gene Co-expression Network Analysis
